# Serum Nardilysin as a Prognostic Biomarker in Pancreatic Ductal Adenocarcinoma

**DOI:** 10.3390/jcm11113101

**Published:** 2022-05-30

**Authors:** Yongfeng Xu, Qunli Xiong, Yang Yang, Ningna Weng, Junjun Li, Jinlu Liu, Xiaojuan Yang, Zhu Zeng, Zhiwei Zhang, Qing Zhu

**Affiliations:** Department of Abdominal Oncology, West China Hospital of Sichuan University, Chengdu 610041, China; hxxyf@stu.scu.edu.cn (Y.X.); 2020224020135@stu.scu.edu.cn (Q.X.); 2019224020123@stu.scu.edu.cn (Y.Y.); 2018324020135@stu.scu.edu.cn (N.W.); 2019324020121@stu.scu.edu.cn (J.L.); liujinlu@stu.scu.edu.cn (J.L.); 2021324025329@stu.scu.edu.cn (X.Y.); 2021324025332@stu.scu.edu.cn (Z.Z.); 2021224020151@stu.scu.edu.cn (Z.Z.)

**Keywords:** nardilysin, serum biomarker, bioinformatics tools, pancreatic ductal adenocarcinoma, carbohydrate antigen 19-9

## Abstract

Background: Nardilysin, (N-arginine dibasic convertase, NRDC) has been reported to play an important role in cancer progression, and is associated with tumor proliferation signals and inflammatory signals, such as tumor necrosis factor-a (TNF-a) and heparin-binding epidermal growth factor-like growth factor (HB-EGF), through the activation of disintegrin and metalloproteinase (ADAM) proteases. NRDC has recently been revealed to be involved in the tumorigenesis of various types of cancer, including intrahepatic cholangiocarcinoma, malignant cerebral infarction, esophageal squamous cell carcinoma, and gastric cancer. However, the expression profiles and biological relevance of NRDC in pancreatic ductal adenocarcinoma have rarely been reported. Methods: We analyzed the NRDC expression profile in pancreatic ductal adenocarcinoma by enzyme-linked immunosorbent assay (ELISA) and identified NRDC as a circulating biomarker in the serum of 112 pancreatic ductal adenocarcinoma patients. The diagnostic value of NRDC was analyzed by the area under the curve (AUC) and the receiver operating characteristic (ROC) test. Results: Our results demonstrated that the clinical prognosis significance of NRDC with the clinical characteristics in pancreatic ductal adenocarcinoma (PDAC). NRDC was notably decreased in PDAC patient serum compared with the control group (*p* < 0.001). Furthermore, the present study found that the NRDC expression level was correlated with T grade (*p* < 0.001), metastasis(*p* < 0.001), differentiation(*p* < 0.001), and TNM stage (*p* = 0.011). Further bioinformatics analysis revealed that NRDC correlated with proliferation and migration pathways; in particular, it mediated cell-matrix adhesion-dependent activation in pancreatic ductal adenocarcinoma. Conclusions: Serum NRDC may play a useful diagnostic biomarker to evaluate the aggressive clinical features in PAAD patients.

## 1. Introduction

Pancreatic ductal adenocarcinoma (PDAC) is characterized by high mortality and poor prognosis [1,2,3]. PDAC accounts for almost as many deaths (466,000) as cases (496,000), and the morbidity and mortality rates are almost equal [4]. The 5-year survival rate at the time of diagnosis is no more than 10% in some European countries [5]. These dismal figures have been attributed to the vagueness of symptoms and a high rate of tumor chemoresistance [6,7,8]. It has been reported that pancreatic ductal adenocarcinoma will replace breast carcinoma as the third leading cause of cancer-related death in Europe by 2025 in a study of 28 European countries [9]. Therefore, we need to find a novel tumor marker to improve the efficiency and accuracy of the early diagnosis of pancreatic ductal adenocarcinoma, improving the survival rate of pancreatic cancer patients.

Nardilysin, N-arginine dibasic convertase, is often abbreviated as NRDC (NRD1, Nrdc), and is a metalloprotease of the M16 family which selectively cleaves at the dibasic terminus of arginine residues [10]. Recently, NRDC has been reported to be valuable in the early diagnosis of intrahepatic cholangiocarcinoma [11], malignant cerebral infarction [12], esophageal squamous carcinoma [13], and gastric cancer [14]. Moreover, it has been reported to be associated with tumor proliferation [15], epithelial–mesenchymal transition (EMT), and inflammatory signals, such as tumor necrosis factor-a (TNF-a), nuclear factor-kappa B(NF-κB), and heparin-binding epidermal growth factor-like growth factor (HB-EGF) [11]. NRDC has also been found in the mitochondria [16], cytoplasm [17], and nucleus [18] which suggests that it might participate in some important signaling pathways. Meanwhile, a report revealed that when NRDC is knocked out in mice, it leads to spontaneous chronic pancreatitis, diabetes, and the stimulation of pancreatic ductal adenocarcinoma initiation [19]. Hence, in order to determine the role of NRDC in pancreatic ductal adenocarcinoma, we designed an observational cohort study to determine the clinical value of NRDC.

## 2. Materials and Methods

### 2.1. Patient Samples

Altogether, 207 participants were under surveillance for this study. Before recruitment, all participants were informed of and signed off the relevant ethical information for this study. Patient blood samples were collected at a median of 24 h before treatment and then centrifuged at 3000× *g* rpm at 4 °C for 10 min. The serum was collected and stored in a −80 °C refrigerator until use. From March 2020 to March 2021, blood samples were collected from all participants before surgery or other therapies at West China Hospital of Sichuan University (Chengdu, China). Pancreatic ductal adenocarcinoma was diagnosed and graded by the Pathology Department of West China Hospital, and TNM staging defined by the American Joint Committee on Cancer (AJCC) criteria standard. After reviewing each patient’s medical history, patients with PDAC who had surgery, a previous history of cancer, were suffering from some inflammatory diseases (for example, pneumonia), or receiving other cancer-related therapies were excluded. This resulted in only 204 patients being enrolled in this study; 112 patients were diagnosed with pancreatic ductal adenocarcinoma (histological verified PDAC) and 92 patients served as the control group (CG), which included 31 benign disease groups (BDG, including intraductal papillary mucinous neoplasm (IPMN), *n* = 6; chronic pancreatitis (CP), *n* = 23; pancreatic neuroendocrine tumor, *n* = 2) and 61 healthy control (HC) patients. Other clinical information, such as grade, height, weight, TNM stage, and the serum level of CA19-9, were all collected from the case management system in West China Hospital.

### 2.2. Cell Lines

Four PDAC cell lines (CAPAN-1, CAPAN-2, BxPC-3, and CFPAC-1) and one normal pancreatic cell line (HPDE) were obtained from the Bei Na Culture Collection (BNCC.org, Chengdu, Sichuan, China). The CAPAN-1 was grown in Iscove’s Modified Dulbecco’s Medium (IMDM, Gibco C12440500BT, New York, NY, USA), CAPAN-2 was grown in Roswell Park Memorial Institute-1640 (RPMI-1640, hyclone SH30809.01, Salt Lake City, UT, USA), BxPC-3 was grown in Dulbecco’s Modified Eagle medium (DMEM, HyClone Cat# SH30243.01, Salt Lake City, UT, USA). The culture media were all supplemented with 10% fetal bovine serum (FBS, Sigma-Aldrich, St. Louis, MO, USA) and penicillin/streptomycin (Meilune, Dalian, Liaoning, China) and cultured in a 5% CO_2_ humidified incubator. Cells were harvested 48 h after incubation and used in qRT-PCR.

### 2.3. Measurement of Serum NRDC Levels

A commercial NRDC ELISA kit (Human Nardilysin ELISA Kit, MyBioSource, Cat. No# MBS1603900, Vancouver, BC, Canada) was used to measure the serum expression level of NRDC. According to the manufacturer’s instructions, the absorbance value was measured on a 96* microplate reader set at a wavelength of 450 nm. The standard curve was plotted to obtain the absolute value of NRDC by comparing it to the standard sample.

### 2.4. RNA Extraction and Quantitative Real-Time PCR (qRT-PCR)

We used TRIzol reagent (Invitrogen, Cat. 10296010, Carlsbad, CA, USA) to extract total RNA from patient specimens and different cell lines (including HPDE, BxPC-3, Capan-1, Capan-2, CFPAC-1). According to the Invitrogen manufacturer’s instructions, we extracted the total RNA, and the quality was determined to be pure only when the A260/A280 ratio was 1.8–2.1 and then reverse-transcribed into cDNA. The qRT-PCR amplification procedure was performed as follows: an initial denaturation at 95 °C for 30 s, followed by 40 cycles at 95 °C for 5 s, 60 °C for 30 s, and 72 °C for 30 s. The related target gene expression was normalized against GAPDH using the 2^−ΔΔCt^ method.

The NRDC primer sequencing results were as follows:

Forward: 5′-GGTCGGTGCGAAGACTCTG-3′

Reverse: 5′-AGATTCATCCGCTCCTAGACG-3′

The GAPDH primer sequencing results were as follows:

Forward: 5′-CTGCACCACCAACTGCTTAG-3′

Reverse: 5′-GTCTTCTGGGTGGCAGTGAT-3′

### 2.5. Immunohistochemistry

Tissue samples were obtained from the pathology platform of West China Hospital and reviewed by the ethics department. After paraffin embedding, the thickness of the tissue samples was sectioned into 3 mm sections deparaffinized in xylene and rehydrated through different concentrations of alcohols (100%, 80%, and 50%). Hydrogen peroxide (3%) was used to block the activity of endogenous peroxidase. A monoclonal anti-NRDC antibody (Proteintech, Cat. No 15630-1-AP, Chicago, IL, USA) was used as the reaction at a dilution of 1:50 overnight at 4 °C. After washing the sections in PBS, we incubated them with biotinylated secondary antibody for 30 min at 37 °C. Diaminobenzidine (DAB) solution was used to color sections as brown staining. For the negative control, 1% BSA/PBS was used in place of primary antibody and was processed in the same manner.

### 2.6. Statistical Analysis

SPSS version 19.0 (IBM Corp, Chicago, IL, USA) was used for all statistical analyses. Because the data were arranged in a non-normal distribution, we utilized nonparametric metrics to assess the differences in serum NRDC and serum CA19-9 expression levels in PDAC patients. The serum NRDC levels were adjusted to account for age and gender variations among the individuals. The respective areas under the curves (AUCs) with a 95% confidence interval (CI), sensitivity, and specificity were evaluated by receiver operating characteristic curves (ROCs). Multivariable logistic regression analysis was used to estimate whether NRDC is an independent factor for TNM staging, and the data were expressed as odds ratios (ORs) and 95% confidence intervals (95% CIs). Finally, we used GraphPad Prism V.8.0 software (GraphPad Software, La Jolla, CA, USA) to draw figures. A *p*-value < 0.05 was considered as a statistically significant difference.

### 2.7. Gene Set Enrichment Analysis

To further explore the underlying mechanisms of the NRDC, GSEA (https://www.gsea-msigdb.org/gsea/index.jsp, accessed on 23 August 2021) was performed in this study. We then divided patient samples into high or low expression groups according to the median expression value of NRDC. The *p*-value < 0.05, FDR < 0.25, and enrichment score (NES) > 1.5 were defined as the significantly enriched gene sets.

### 2.8. Gene Ontology (GO), Kyoto Encyclopedia of Genes and Genomes (KEGG) Analysis, Heatmap, and Volcano Plots

GO and KEGG enrichment studies were carried out in R language using the programs clusterProfiler, enrichplot, and ggplot2. Only results with *p*-values and q-values no more than 0.05 were deemed significantly enriched. The heatmap and volcano plots of NRDC were produced by R language, and only logFC > 0.5 and *p*-value < 0.05 were treated as significant.

### 2.9. Pan-Cancer Analysis

UCSC Xena (http://xena.ucsc.edu/, accessed on 26 August 2021) is a multicancer type database that provides data analysis for a variety of tumors, including multicancer gene expression levels, polygene analysis, and multi-cancer research database functions, providing visual analysis for public data centers. The expression row data were downloaded from UCSC and analyzed by R software; *p*-values less than 0.05 were considered as statistically significant differences.

## 3. Results

### 3.1. Participants’ Characteristics

According to our inclusion and discharge criteria, there were 112 PDAC (pancreatic ductal adenocarcinoma) patients and 92 non-PDAC patients (HC, healthy control, *n* = 61; BDG, benign disease group, including CP, chronic pancreatitis, *n* = 23; PNT, pancreatic neuroendocrine tumor, *n* = 2; IPMN, intraductal papillary mucinous neoplasm, *n* = 6). The average age was 60 years old (25–80 years old), and most of the test subjects were male (65.1%). Among 112 PDAC patients, the ages ranged from 37 to 81 years. According to AJCC stages, patients with pancreatic ductal adenocarcinoma were divided into four groups: stage I patients (*n* = 34), stage II patients (*n* = 30), stage III patients (*n* = 18), and stage IV patients (*n* = 30). The degree of differentiation, distant metastasis, and other clinical characteristics of the patients were analyzed by the χ^2^ test and are shown in Table 1, and NRDC expression in the PDAC (771 pg/mL) was significantly lower than that in most other lesions combined (1368 pg/mL), IPMN (1308 pg/mL), or CP (1132 pg/mL) (*p* < 0.0001, *p* = 0.0054, and *p* = 0.0006, respectively, Supplementary Table S1). After the χ^2^ test, we found that the expression of NRDC was significantly associated with TNM stage (*p* = 0.011), differentiation (*p* < 0.001), metastasis (*p* < 0.001), and tumor size (*p* < 0.001). However, there was no significant (NS) correlation of NRDC expression with other clinical features, such as sex, age, drinking, and smoking (*p* > 0.05).

### 3.2. The Relative Level of NRDC Expression in Serum, Cell Lines or Pancreatic Ductal Adenocarcinoma Tissues

**Figure 1:**
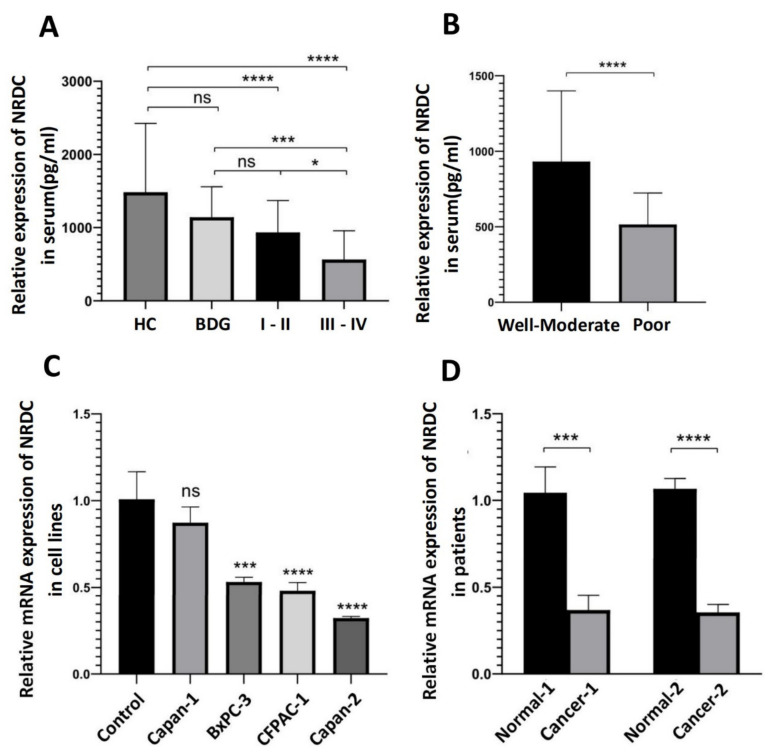
The expression levels of NRDC. (**A**) Serum NRDC levels in control group and PDAC different staging groups. HC (*n* = 61), BDG (*n* = 31), and PDAC including I/II (*n* = 63), III/IV (*n* = 48). (**B**) Serum NRDC levels in various differentiation groups, well-moderated group (*n* = 58), poor differentiation group (*n* = 25). (**C**) Relative mRNA expression levels in cell lines, all PDAC cell lines were compared to the normal pancreatic cell lines. The procedure was repeated three times, consistent with the previous results. (**D**) Relative mRNA expression levels in two patients, Cancer-1/2 were from two different patients’ cancer tissues, Normal-1/2 were from patients’ peritumoral tissues. The procedure was repeated three times in six patients’ tissues, consistent with the previous results. * *p* < 0.05, *** *p* < 0.001, and **** *p* < 0.0001. ns: none significance, HC: healthy control, BDG: benign disease group.

Consequently, we wondered whether NRDC is an excellent tumor marker to evaluate the TNM staging and histopathological grading of pancreatic ductal adenocarcinoma. NRDC expression was significantly decreased in stage III/IV PDAC compared with stage I/II PDAC and the control group (Figure 1A). Moreover, compared with the well-moderate differentiation group, the poor differentiation group had a significantly lower serum NRDC level (Figure 1B). We then analyzed the significant differences between the benign disease group (BDG) and the tumor/healthy group by an unpaired *t*-test (due to the limitation of the number of pancreatic neuroendocrine tumor we only analyzed the rest of groups, and the results of the comparison between the groups as is shown in Supplementary Figure S1). In order to verify the NRDC transcriptional level in pancreatic ductal adenocarcinoma cell lines. A normal pancreatic cell line, HPDE, was treated as a control group for pancreatic ductal adenocarcinoma cell lines to measure the transcriptional level of NRDC by qRT-PCR. Compared with the control group, the expression of NRDC was decreased in different pancreatic ductal adenocarcinoma cell lines (Figure 1C). Similarly, we repeated the previous qRT-PCR procedure and the same conclusion was observed in the patient tissues (Figure 1D). In other words, the expression of NRDC may be negatively correlated with the clinical stage and grade in pancreatic ductal adenocarcinoma.

### 3.3. Diagnostic Value of Serum NRDC and CA19-9

**Figure 2:**
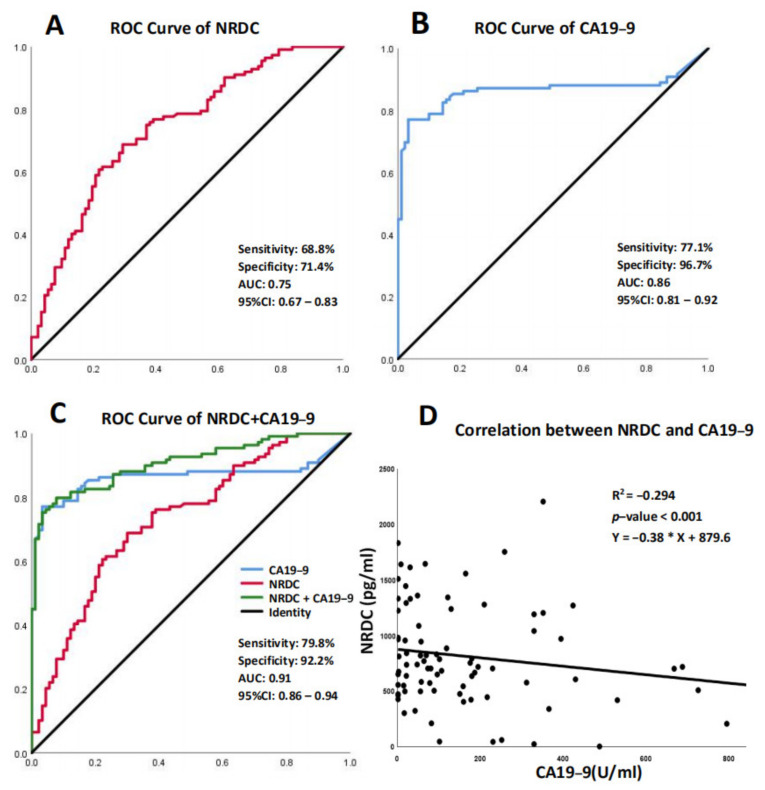
Receiver operating characteristic curve (ROC) analyses of serum NRDC and CA19-9 levels in the diagnosis of PDAC. (**A**) ROC curve of NRDC. (**B**) ROC curve of CA19-9. (**C**) ROC curve of NRDC, CA19-9 and combined group, the sensitivity, specificity, AUC and CI of combined group were shown. (**D**) Correlation analysis between NRDC and CA19-9 in PDAC patient.

Carbohydrate antigen 19-9 (CA19-9) is one of the most popular serum biomarkers for the diagnosis of PDAC in the clinic. Hence, we measured NRDC expression in the experimental and control groups to compare sensitivity and specificity with CA19-9. Receiver operating characteristic (ROC) curves were constructed to prove the relationship between cutoff value, sensitivity and specificity, and curves were constructed between PDAC and controls. The defined optimum cutoff point of NRDC was 865 pg/mL and the optimum cutoff point of CA19-9 was 40.5 U/mL. Therefore, according to the cutoff point, we chose 865 pg/mL as a critical value to determine whether the patient’s serum level of NRDC was highly expressed. The positive predictive value (PPV), negative predictive value (NPV), and accuracy are as follows. NRDC: PPV:74.8%, NPV: 65.3%, accuracy: 70.1%. CA19-9: PPV: 95.6%, NPV: 77.2%, accuracy: 85.3%. Combined group: PPV: 92.7%, NPV: 88.0%, accuracy: 85.3%. The values of the area under the curve (AUC) of NRDC versus CA19-9 were 0.74 (95% CI: 0.67–0.83, sensitivity and specificity were 68.8% and 71.4%, respectively) and 0.86 (95% CI: 0.81–0.92, sensitivity and specificity were 77.1% and 96.7%, respectively; Figure 2A,B).

Next, this study assessed the diagnostic performance of NRDC in combination with CA19-9 and analyzed the possibility of combining NRDC with CA19-9 both in controls and in PDAC patients. When the AUC of CA19-9 and NRDC (combined group) were compared, the AUC for the combined group was higher than the CA19-9 group (0.907, 95% CI: 0.867–0.948 vs. 0.86, 95% CI: 0.806–0.922). The sensitivity and specificity of the combined group vs. the CA19-9 group were as follows: 79.8% vs. 77.1% and 92.2% vs. 96.7% (Figure 2C). These data showed that the combination of NRDC with CA19-9 could promote the diagnostic power of CA19-9 in PDAC patients, suggesting that their combination serves as a potential biomarker for PDAC diagnosis. Furthermore, we investigated the correlation between NRDC and CA19-9. Due to the limitations of the test kit, the maximum measured value of CA19-9 was 1000 U/mL, and results greater than or equal to 1000 U/mL will be ignored. Next, correlations of NRDC levels with CA19-9 levels were assessed by Spearman’s correlation analysis in all patients, and we found a trend of negative correlation between their levels (R^2^ = −0.294, *p*-value < 0.001, Figure 2D).

### 3.4. Immunohistochemistry (IHC) Staining of NRDC and Hematoxylin-Eosin (H&E) Staining in Human Pancreatic Ductal Adenocarcinoma Tissues

**Figure 3:**
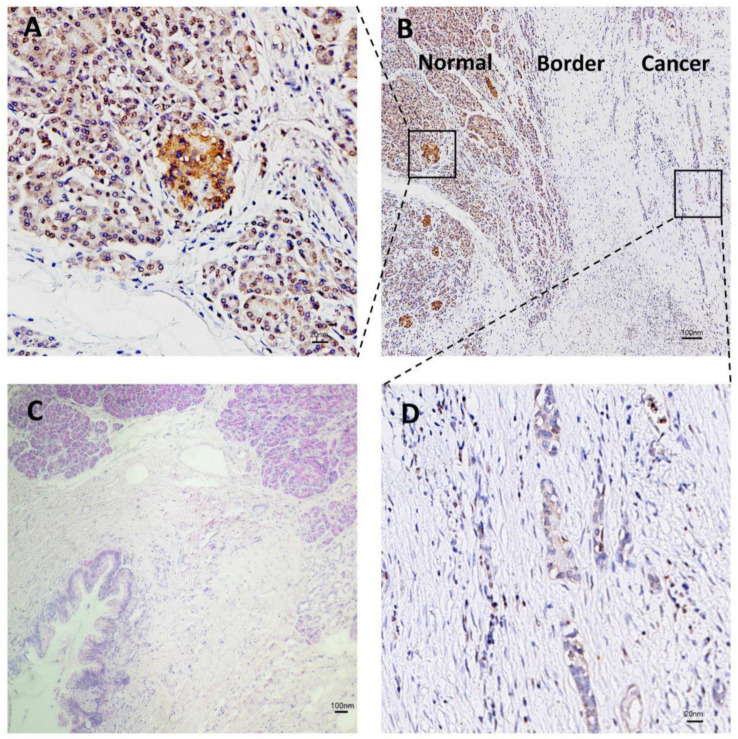
Immunohistochemistry (IHC) staining tissues of NRDC in pancreatic ductal adenocarcinoma. (**A**) Expression of NRDC protein in human pancreatic normal tissue (*n* = 6). (**B**) The overview of IHC in cancer and normal tissue. (**C**) H&E staining of human pancreatic ductal adenocarcinoma and normal tissue. (**D**) Expression of NRDC protein in human pancreatic ductal adenocarcinoma tissue (*n* = 9).

To determine the prevalence and clinical significance of NRDC in PDAC tissue, we assessed the performance of NRDC detection in patients’ pancreas by immunohistochemistry. Compared with cancer tissue, the expression level of NRDC is highly expressed in normal tissue (Figure 3A,B,D). H&E staining was performed on both cancer and normal tissues (Figure 3C).

### 3.5. Gene Set Enrichment Analysis (GSEA), Heatmap and Volcano Plot

**Figure 4:**
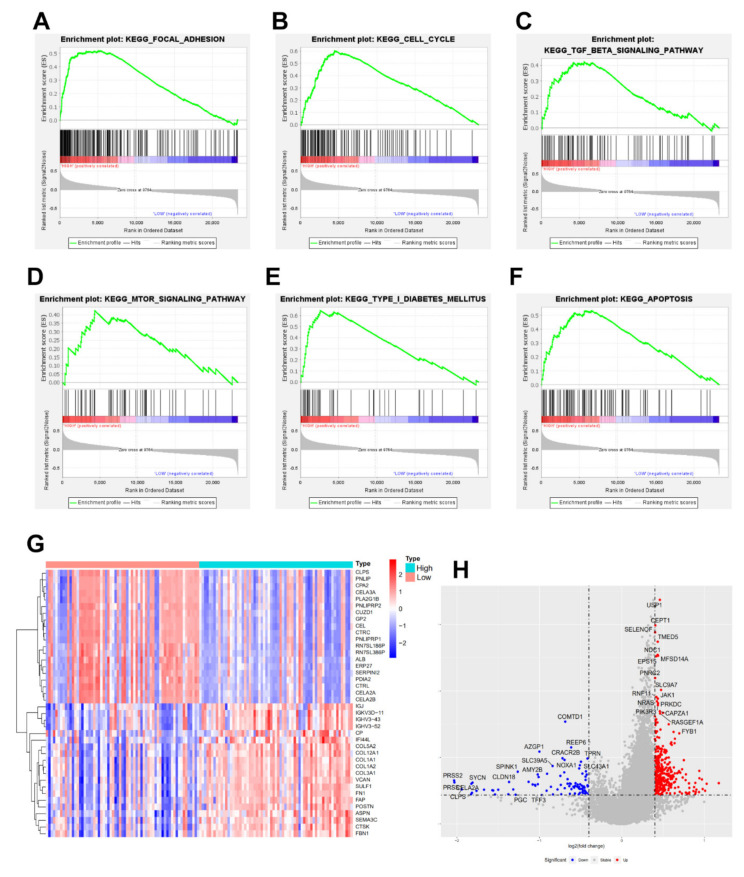
Gene set enrichment analysis (GSEA) of NRDC, (**A**–**F**) different signal pathway enrichment of NRDC, curves marked with green flag mean gene enrichment. Peaks on the upward curve indicate positive correlation. (**G**) according to the average expression of NRDC, divided genes into high and low groups, heatmap of the top 40 differentially expressed genes in the analysis result (Red: up-regulation; Blue: down-regulation), (**H**) divided the expression of NRDC into high and low groups, volcano plot of differential genes were expressed (logFC ≥ 0.4 and −log10 (adj. *p* Value) ≥ 1 were defined as significance. Red: up-regulation; Blue: down-regulation).

To further explore the underlying mechanism, we constructed GSEA based on the expression of NRDC; all bioinformatics analyses were sourced from TCGA, GEO (GSE102238, GSE62452), and University of California Santa Cruz (UCSC). As expected, NRDC was enriched in a number of important signaling pathways, such as focal adhesion, cell cycle, proliferation, autophagy, type I diabetes, and apoptosis pathway activation in pancreatic ductal adenocarcinoma (Figure 4A–F). Given that the levels of NRDC were negatively correlated with the differentiation and stage of PDAC patients, we separated patients into high-expression and low-expression groups compared with the median level of NRDC expression. The heatmap and volcano plot are exhibited in Figure 4G,H, respectively. As shown in the plots, multiple genes changed consistently with the expression of NRDC. These results suggested that NRDC might play a potential role in interacting with many proteins.

### 3.6. Gene Ontology (GO) Analysis, Kyoto Encyclopedia of Genes and Genomes (KEGG) Analysis, and Pan-Cancer Analysis of NRDC

**Figure 5:**
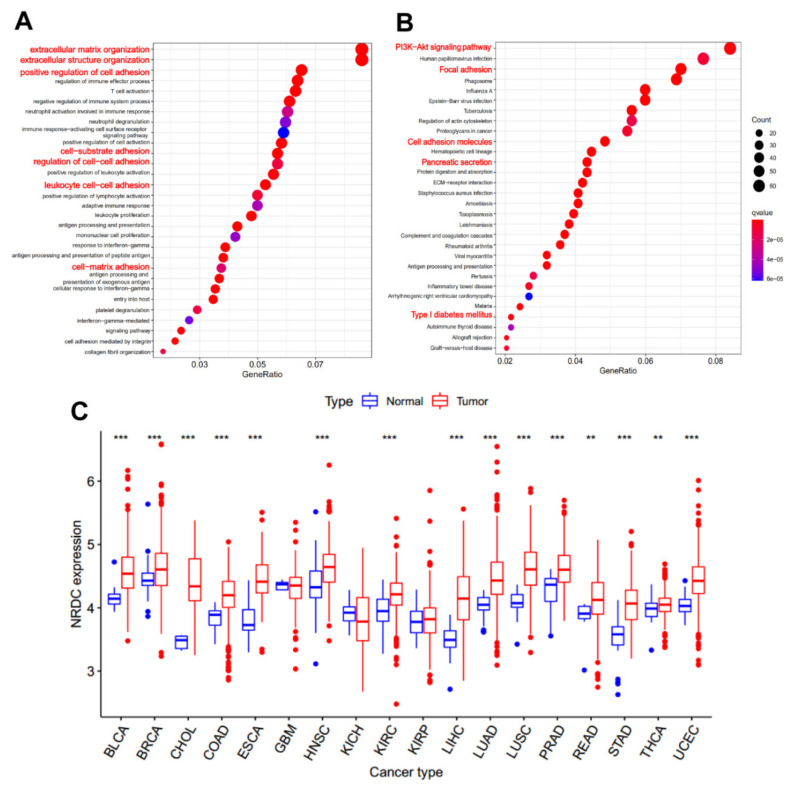
Results of gene ontology (GO) enrichment analysis of NRDC in GSE102238. (**A**) Biological process categories of enriched terms. (**B**) Kyoto Encyclopedia of Genes and Genomes (KEGG) pathway analysis of NRDC, printed by *p*-values and gene counts. (**C**) The expression of NRDC in different cancer types. Bar plots profiling gene expression between tumor samples (red) and paired normal samples (blue). (BLCA: bladder urothelial carcinoma; BRCA: breast invasive carcinoma; CHOL: cholangiocarcinoma, COAD: colon adenocarcinoma; ESCA: esophageal carcinoma; GBM: glioblastoma multiforme; HNSC: head and neck squamous cell carcinoma; KICH: kidney chromophobe; KIRC: kidney renal clear cell carcinoma; KIRP: kidney renal papillary cell carcinoma; LIHC: liver hepatocellular carcinoma; LUAD: lung adenocarcinoma; LUSC: lung squamous cell carcinoma; PRAD: prostate adenocarcinoma; READ: rectum adenocarcinoma; STAD: stomach adenocarcinoma; THCA: thyroid carcinoma; UCEC: uterine corpus endometrial carcinoma; **: *p* < 0.01; ***: *p* < 0.001).

After knowing the possible role of NRDC in the pancreas, we began to explore the function of NRDC, which may play a potential role in PDAC development. The Gene Ontology (GO) analysis showed that NRDC was significantly associated with extracellular matrix organization, cell-cell adhesion, cell-matrix adhesion, regulation of the stability of the extracellular matrix, and so on (Figure 5A). Kyoto Encyclopedia of Genes and Genomes (KEGG) examination showed that NRDC is enriched in several interesting pathways, such as focal adhesion, PI3K-Akt pathway, pancreatic secretion, and type I diabetes (Figure 5B). Although a significant effect of NRDC on pancreatic ductal adenocarcinoma pathobiology is evident, its clinical significance in other cancer types remains poorly elucidated. Hence, we used the University of California Santa Cruz (UCSC) Genome Browser database to expound the expression levels in other different cancer types (Figure 5C).

## 4. Discussion

Pancreatic ductal adenocarcinoma is known for its extremely high mortality and the overwhelming majority of patients with locally advanced or distant metastasis (80–85%). The majority of patients are unresectable (75–80%) [20,21,22,23] and have a low 5-year overall survival rate [24,25,26]. CA19-9 is not recommended for screening early pancreatic cancer because of its limited sensitivity [27,28,29], especially in small tumors [30]. Hence, detection for early-stage PDAC and the evaluation of PDAC prognosis remain difficult problems.

Previous functional studies provided evidence that NRDC is a tumor-associated protein that is upregulated in numerous human cancers, including intrahepatic cholangiocarcinoma [11], esophageal squamous cell carcinoma [13], and gastric cancer [14], and is associated with clinical characteristics, suggesting important roles for NRDC in tumor biology. NRDC is distributed throughout the body and affects a variety of biological functions, including inflammation [31], body temperature homeostasis [32], and insulin secretion [18]. Moreover, although NRDC is a soluble cytosolic protein, it shuttles between the cytoplasm and nucleus by an unknown mechanism [33] and plays a potential drug role in antiangiogenic therapy [34]. Some studies also identified NRDC, a metalloendopeptidase, as a specific binding partner of heparin-binding epidermal growth factor-like growth factor (HB-EGF) [11]. Metalloendopeptidase is a transmembrane glycoprotein that degrades a number of substrates; it has been linked to cancer metastasis [35], angiogenesis [36,37], and modifications in adhesion results in the development of adenocarcinomas that incline to invade vessels and form proliferative entities [37,38]. In addition, previous reports indicated that NRDC was highly expressed in many other types of tumors [11,13,14]. Yoh et al., reported that NRDC is highly expressed in cholangiocarcinoma [11], and this is the opposite of what we found in pancreatic cancer. Interestingly, sometimes it is difficult for clinicians and pathologists to distinguish between distal cholangiocarcinoma and pancreatic head cancer, so NRDC may play a potential role in identifying them. Moreover, there are still some reports indicating that deletion of NRDC in the mice’s pancreas dramatically leads to spontaneous chronic pancreatitis and accelerates KRAS-driven pancreatic tumorigenesis [19], demonstrating that NRDC may play a kind of tumor-suppressive function and be an essential factor in the growth in the pancreas.

Therefore, in the present research, we identified that NRDC expression was significantly decreased in the serum of 112 patients compared with the healthy group. Moreover, we found that the expression of NRDC was negatively correlated with tumor size, metastasis, differentiation, and TNM staging in patients. Furthermore, RT-PCR and immunohistochemistry demonstrated that NRDC was not only decreased in different pancreatic ductal adenocarcinoma cell lines but also decreased in patients’ PDAC tissues. Meanwhile, NRDC could discriminate PDAC from the control group with an AUC of 0.75 (95% CI: 0.67–0.83). These findings provided evidence for detecting PDAC by using NRDC as a novel marker to estimate metastasis, grading, differentiation, and diagnosis. NRDC may play an important role in estimating metastasis, grading, and differentiation (*p* < 0.0001) in PDAC. However, our study still has several limitations. The specificity of CA19-9 is 96.7% in our results, which is greater than the range we anticipated, i.e., from 68% to 80% [39]. It might be that the majority of normal patients (87/90, 96.67%) had serum CA19-9 levels that fall in the normal range. Moreover, due to the short follow-up time, we were unable to obtain an accurate median survival time and perform a Kaplan–Meier test. Additionally, experiments in vitro and in vivo were deemed necessary to further confirm the biological activity of NRDC in pancreatic ductal adenocarcinoma. Understanding the underlying mechanism of NRDC in PDAC will be helpful to establishing a novel diagnostic biomarker.

## Figures and Tables

**Table 1:** Characteristics of patients with pancreatic ductal adenocarcinoma.

Characteristics	Number	NRDC		χ^2^	*p*-Value
		Low	High		
Age (years)				0.913	NS
<60	47 (42)	30 (64)	17 (36)		
≥60	65 (58)	47 (72)	18 (28)		
Gender				2.662	NS
Male	73 (57)	54 (57)	19 (43)		
Female	39 (43)	23 (43)	16 (57)		
Smoke				0.018	NS
Yes	67 (60)	47 (70)	20 (30)		
No	35 (40)	25 (71)	10 (29)		
Drink				0.492	NS
Yes	77 (75)	55 (71)	22 (29)		
No	25 (25)	16 (64)	9 (36)		
Metastasis				30.915	<0.001
Yes	26 (25)	23 (88)	3 (12)		
No	76 (75)	47 (62)	29 (38)		
Differentiation				28.221	<0.001
Well-Moderate	57 (51)	32 (56)	25 (44)		
Poor Missing	24 (21)	23 (96)	1 (4)		
	31 (28)				
T stage				26.958	<0.001
T1	4 (4)	2 (50)	2 (50)		
T2	45 (40)	30 (67)	15 (33)		
T3	1312)	8 (62)	5 (38)		
T4 Missing	40 (36)	10 (25)	30 (75)		
	10 (8)				
TNM stage				6.4	=0.011
I/II	63 (56)	37 (65)	26 (35)		
III/IV Missing	48 (43)	39 (81)	9 (19)		
	1 (1)				

NS: no significance.

## Data Availability

The dataset generated for this study are available on request to the corresponding author.

## Supplementary Materials

The following supporting information can be downloaded at: https://www.mdpi.com/article/10.3390/jcm11113101/s1, Figure S1: Relative expression of serum NRDC in different subgroups; Table S1: Expression of NRDC in PDAC and its precursor lesions.
