# Supplementary material for: Serum Nardilysin as a Prognostic Biomarker in Pancreatic Ductal Adenocarcinoma
Source: J Clin Med. 2022 May 30;11(11):3101. doi: 10.3390/jcm11113101 (PMC9181681; doi:10.3390/jcm11113101)
Supplement: Supplementary file 1 [file jcm-11-03101-s001.zip › jcm-1709406-supplementary.pdf]

# Supplementary Figure S1

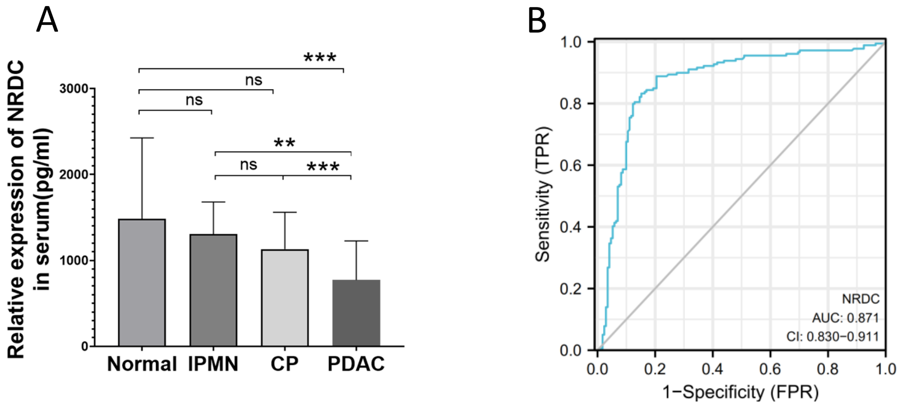


**Figure S1**. The expression levels of NRDC in different groups, including : (**A**) intraductal papillary mucinous neoplasm(IPMN) : n=6, chronic pancreatitis(CP) : n=23, pancreatic neuroendocrine tumor(PN) : n=2, Normal Control(NC):61, pancreatic ductal adenocarcinoma(PDAC) : n=112 . (**B**) Receiver operating characteristic curve (ROC) analyses of serum NRDC in TCGA and GTEx database. ns: none significance, ** $p < 0.01$, *** $p < 0.001$.

# Supplementary Table S1

**Table S1.** Expression of NRDC in PDAC and its precursor lesions.

| Diagnosis | *n* | Expression score mean ± SD | *P* value |
|---|---|---|---|
| intraductal papillary mucinous neoplasm(IPMN) | 6 | 1308 ± 340 | *p*=0.0054 (vs PDAC) |
| chronic pancreatitis(CP) | 23 | 1132 ± 418 | *p*=0.0006 (vs PDAC) |
| pancreatic neuroendocrine tumor(PNT) | 2 | 747 ± 66 | *p*=0.93965 (vs PDAC) |
| Normal Control(NC) | 61 | 1483 ± 933 | *p*<0.0001 (vs PDAC) |
| pancreatic ductal adenocarcinoma(PDAC) | 112 | 771 ± 453 | *p*<0.0001 (vs others) |

SD, standard deviation; vs, versus
